# Intravesical therapy for bladder cancer causing disseminated tuberculosis and perforated viscus: a case report

**DOI:** 10.1093/jscr/rjag131

**Published:** 2026-03-12

**Authors:** Wed Alwabel, Abdulrahman Almisfer, Ibrahim Alsamaani, Bader Aljaafri, Nayef Alzahrani

**Affiliations:** College of Medicine, King Saud bin Abdulaziz University for Health Sciences, Riyadh, Saudi Arabia; Department of Surgery, Ministry National Guard Health Affairs, King Abdulaziz Medical City, Riyadh, Saudi Arabia; Department of Surgery, Ministry National Guard Health Affairs, King Abdulaziz Medical City, Riyadh, Saudi Arabia; Department of Surgery, Ministry National Guard Health Affairs, King Abdulaziz Medical City, Riyadh, Saudi Arabia; Department of Surgery, Ministry National Guard Health Affairs, King Abdulaziz Medical City, Riyadh, Saudi Arabia

**Keywords:** bladder cancer, disseminated tuberculosis, abdominal tuberculosis, bacillus Calmette-Guérin, bowel perforation, ileocecal perforation

## Abstract

The ileocecal area and peritoneum are frequently affected by abdominal TB, which is uncommon (1%–3%) and usually manifests as vague symptoms that resemble malignancies. A 6–9 months of antituberculous medication is the standard course of treatment; surgery is saved for complications like obstruction or perforation. We present a case of an 81-year-old man who had recurrent bladder cancer and had previously intravesical Bacillus Calmette-Guérin (BCG) treatment. He developed diarrhea, vomiting, and a small intestinal perforation in 2025, which were originally suspected to be signs of metastatic dissemination. Necrotizing granulomatous inflammation was identified during ileocecal resection, and testing verified disseminated tuberculosis, which is most compatible with systemic BCG infection.

## Introduction

Tuberculosis (TB) remains the leading cause of death from a single infectious agent worldwide [1]. Abdominal TB is a form of extrapulmonary disease representing 1%–3% of all TB cases and up to 13% of extrapulmonary TB. It may involve the gastrointestinal tract, peritoneum, solid organs, or intra-abdominal lymph nodes, with the ileum, ileocecal region, and peritoneum being the most commonly affected sites [1].

Clinical presentation is often nonspecific, including abdominal pain, anorexia, weight loss, diarrheoa, or fever, frequently overlapping with malignancy, inflammatory bowel disease, or other infections. Diagnosis relies on a combination of imaging, endoscopy, microbiologic testing, and histopathology. Standard therapy consists of antituberculous drugs for 6–9 months, while surgery is reserved for complications such as obstruction, perforation, or failure of medical management [2].

## Case report

An 81-year-old male known to have non-invasive papillary urothelial carcinoma of the bladder. He underwent transurethral resection of bladder tumour in 2015 and was planned for future follow ups and surveillance. He missed his follow up appointments for 2 years. In 2018 he attended clinic follow up where his primary urologist ordered renal ultrasound which showed small urinary bladder polyps. He was booked for urgent cystoscopy and a second transurethral resection of bladder tumour (TURBT) was done with tissue pathology showing low-grade papillary urothelial carcinoma. He was planned for six cycles of intravesical mitomycin which he completed and was again put back on surveillance.

In 2020 during routine cystoscopy, multiple masses seen which were concerning for recurrence. A third TURBT was done and tissue pathology showed high grade papillary urothelial carcinoma. Another course of mitomycin was started along with intravesical Bacille Calmette–Guérin (BCG). He completed the course in 2021 and was doing well on routine follow ups with no signs of recurrence.

**Figure 1:**
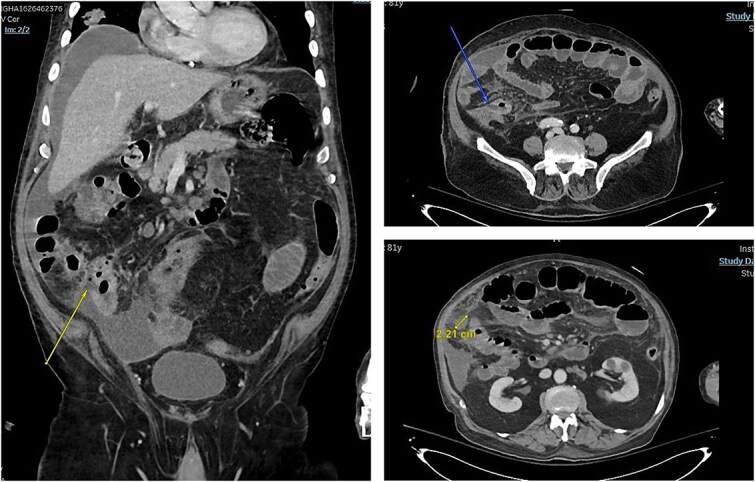
Arrows pointing towards areas of bowel wall defect.

In 2025, he presented to the emergency department (ED) with a 3-week history of persistent diarrhea, lethargy, and vomiting. At presentation, he had no features of active pneumonia and was on room air. Abdominal computed tomography (CT) was done and it showed partial small bowel obstruction with perforated terminal ileum, could be related to underlying ischemia or metastatic involvement. Peritoneal fluid can be appreciated in CT scan (Fig. 1).

Overall findings were in keeping with progressive metastatic disease involving the peritoneum, lymph nodes, liver, and lungs. Family meeting was done to discuss our plan which was urgent surgery. He was taken for exploratory laparotomy, ileocecal resection, and end ileostomy with abdominal washout. Post operatively he was kept in the intensive care unit (ICU) where they did CT chest since he was difficult to wean off oxygen and it showed - multiple bilateral lung nodules and masses with enlarged intrathoracic lymph nodes, considering patient history of bladder cancer and recent CT abdomen findings, chest findings are in keeping with metastasis.

The ICU team sent respiratory samples for tuberculosis (TB) workup, which returned positive, supporting disseminated mycobacterial infection and possibly contributing to his postoperative respiratory deterioration and oxygen requirement. Histopathological examination of the resected bowel further confirmed the diagnosis, demonstrating necrotizing granulomatous enterocolitis secondary to Mycobacterium tuberculosis infection.

Despite Anti TB treatment, he continued to deteriorate in terms of TB pneumonia, intra-abdominal collection, and bone marrow suppression. He ended up with septic shock and mutli-organ failure which both infectious disease team and ICU team agreed that it is primarily due to his disseminated TB. A month later from his surgery he passed away due to his sepsis in ICU.

## Discussion

Intravesical BCG is an established adjuvant therapy for intermediate and high risk non- invasive bladder cancer and is recommended by international guidelines. Its antitumour effect is mediated through immune activation and localized inflammatory responses within the bladder. Although generally safe, adverse effects occur, most commonly local cystitis-like symptoms and mild systemic reactions [3].

Disseminated BCG infection, sometimes referred to as BCGosis, is rare but potentially fatal. Reported incidence is ~1%, with extrapulmonary involvement being more common than pulmonary disease. Presentations may occur months to years after treatment, and late-onset cases several years after the last instillation have been described [4].

Risk factors include mucosal disruption during catheterization or recent transurethral procedures, advanced age, immunosuppression, and active urinary tract infection. Advanced age has been associated with higher mortality in disseminated disease, likely reflecting reduced physiologic reserve and impaired immune response [5, 6].

In this case, the patient’s presentation with bowel perforation and radiologic features suggestive of metastatic cancer highlights the diagnostic difficulty of abdominal TB and disseminated BCG infection. Furthermore, he had several risk factors predisposing to disseminated tuberculosis following intravesical BCG therapy. Advanced age is a well-established risk factor and is associated with impaired immune response and higher mortality in disseminated BCG infection. Repeated transurethral resections of bladder tumor may have caused cumulative urothelial disruption, facilitating systemic absorption of BCG. In addition, high-grade urothelial carcinoma and prior intravesical chemotherapy may have contributed to altered immune surveillance. In addition, the prolonged interval of 4 years after BCG therapy raises the possibility of de novo Mycobacterium tuberculosis infection, particularly in TB-endemic regions, so definitive differentiation requires mycobacterial culture or molecular speciation to identify *Mycobacterium bovis* (BCG strain), which is intrinsically resistant to pyrazinamide [7].

Failure to respond to therapy in this patient may be explained by delayed diagnosis, extensive multisystem involvement, postoperative stress with concurrent intra-abdominal sepsis, and potential suboptimal therapy if the causative organism was *M. bovis*. These factors likely contributed to progression to septic shock and death despite appropriate multidisciplinary management.

## Conclusion

Disseminated mycobacterial infection following intravesical BCG therapy is rare but carries a high mortality, particularly in elderly patients presenting with severe systemic illness. This case underscores the importance of considering mycobacterial infection in patients with prior BCG exposure who develop granulomatous disease or unexplained systemic deterioration. Early recognition, species-level identification, and prompt initiation of appropriate therapy are essential, although outcomes may remain poor in patients presenting with advanced disease and surgical complications.

## References

[1] Janssen S, Murphy M, Upton C et al. Tuberculosis: an update for the clinician. Respirology (Carlton, Vic) 2025;30:196–205. 10.1111/resp.14887 39887565 PMC11872285

[2] Tobin EH, Khatri AM. Abdominal Tuberculosis. In: StatPearls Publishing LLC (eds.) StatPearls [Internet]. Treasure Island (FL): StatPearls Publishing, 2025. Available from: https://www.ncbi.nlm.nih.gov/books/NBK556115/. 32310575

[3] Babjuk M, Burger M, Capoun O et al. European Association of Urology guidelines on non–muscle-invasive bladder cancer (Ta, T1, and carcinoma in situ). Eur Urol 2022;81:75–94. 10.1016/j.eururo.2021.08.010 34511303

[4] Lamm DL, Stogdill VD, Stogdill BJ et al. Complications of Bacillus Calmette-Guerin immunotherapy in 1,278 patients with bladder cancer. J Urol 1986;135:272–4. 3511286 10.1016/s0022-5347(17)45606-0

[5] Asín MA, Fernández-Ruiz M, López-Medrano F et al. Bacillus Calmette-Guérin (BCG) infection following intravesical BCG administration as adjunctive therapy for bladder cancer: incidence, risk factors, and outcome in a single-institution series and review of the literature. Medicine 2014;93:236–54. 25398060 10.1097/MD.0000000000000119 PMC4602419

[6] Decaestecker K, Oosterlinck W. Managing the adverse events of intravesical Bacillus Calmette–Guérin therapy. Res Rep Urol 2015;7:157–63. 10.2147/RRU.S63448 26605208 PMC4630183

[7] de Jong BC, Onipede A, Pym AS et al. Does resistance to pyrazinamide accurately indicate the presence of *Mycobacterium bovis*? J Clin Microbiol 2005;43:3530–2. 16000498 10.1128/JCM.43.7.3530-3532.2005 PMC1169118

